# Sepsis auf deutschen Intensivstationen – Weltweit Schlusslicht? … Nicht so schnell

**DOI:** 10.1007/s00101-021-00986-9

**Published:** 2021-06-21

**Authors:** Michael Bauer, Heinrich Volker Groesdonk, Franziska Preissing, Petra Dickmann, Tobias Vogelmann, Herwig Gerlach

**Affiliations:** 1 Klinik für Anästhesiologie und Intensivmedizin, Universitätsklinikum Jena, Am Klinikum 1, 07747 Jena, Deutschland; 2 Klinik für Interdisziplinäre Intensivmedizin und Intermediate Care, Helios Klinikum Erfurt, Nordhäuser Straße 74, 99089 Erfurt, Deutschland; 3 CytoSorbents Europe GmbH, Müggelseedamm 131, 12587 Berlin, Deutschland; 4 LinkCare GmbH, Kyffhäuserstr. 64, 70469 Stuttgart, Deutschland; 5 Klinik für Anästhesie, operative Intensivmedizin und Schmerztherapie, Vivantes Klinikum Neukölln, Rudower Straße 48, 12351 Berlin, Deutschland

## Weitere Artikel zu diesem Beitrag

**Editorial.**

Briegel J, Brenner Th (2021) Sterblichkeit bei Sepsis in Deutschland – Eine Frage der Repräsentativität! *Anaesthesist. *10.1007/s00101-021-00984-x [gedruckt in Ausgabe 08/2021]

**Originalartikel.**

Bauer M. Groesdonk HV, Preissing F et al (2021) Sterblichkeit bei Sepsis und septischem Schock in Deutschland. Ergebnisse eines systematischen Reviews mit Metaanalyse. *Anaesthesist*. 10.1007/s00101-021-00917-8 [gedruckt in Ausgabe 08/2021]

**Leserbriefe.**

Gründling M, Kuhn S‑O, Scheer C (2021) Sepsisletalität in Deutschland – gute vergleichbare Daten sind aktuell nicht verfügbar. *Anaesthesist.* 10.1007/s00101-021-00987-8 [gedruckt in Ausgabe 08/2021]

Reinhart K (2021) Methodische Schwächen und Fehler bei der Datenextraktion führen zu einer erheblichen Unterschätzung der Sterblichkeit bei Sepsis und septischem Schock in Deutschland. *Anaesthesist.*. 10.1007/s00101-021-00988-7 [gedruckt in Ausgabe 08/2021]

**Erratum.**

Bauer M, Groesdonk HV, Preissing F et al (2021) Erratum zu: Sterblichkeit bei Sepsis und septischem Schock in Deutschland. Ergebnisse eines systematischen Reviews mit Metaanalyse. *Anaesthesist *(2021). 10.1007/s00101-021-00985-w [online auf www.springermedizin.de]

Die Autoren danken Herrn Reinhart und der Arbeitsgruppe um Matthias Gründling für die intensive und kritische Beschäftigung mit unserer Arbeit. Beide Autoren adressieren grundsätzlich zwei Themenkomplexe, nämlich methodische Aspekte und die Frage eines potenziellen Interessenkonflikts, sodass im Folgenden eine gemeinsame Diskussion beider Zuschriften in der geschilderten Reihenfolge der Themen erfolgen soll:

Beide Leserbriefe weisen zutreffend darauf hin, dass es sich bei den in unsere Metaanalyse [1] eingeschlossenen Studien um 6 randomisiert kontrollierte Studien (RCT) zur Untersuchung von pharmakologischen und medizintechnischen Interventionen handelte, 8 Studien waren prospektive Beobachtungsstudien (davon untersuchten 2 den Einfluss von Qualitätsinitiativen), und eine Studie war eine retrospektive Beobachtungsstudie. Die 28-Tages-/30-Tages-Sterblichkeit von Sepsis in Metaanalysen anhand derartiger Studien zu untersuchen, ist internationaler Standard, wie die zuletzt auf RCT basierenden Metaanalysen zu genau dieser Frage von Stevenson et al. [2] und Luhr et al. [3] zeigen. Wir stimmen den Leserbriefschreibern zu, dass mehr Daten zu 28-Tages- und 90-Tages-Letalität aus der Versorgungsrealität verschiedener Länder wünschenswert wären. Diese liegen in dieser Form allerdings nicht vor, wie unsere Metaanalyse auch gezeigt hat. Ein internationaler Vergleich muss sich daher auf die verfügbare Evidenz stützen.

Herr Reinhart weist darauf hin, dass Studien, die die Krankenhaussterblichkeit untersuchten, in der Metaanalyse unberücksichtigt blieben. Das Outcome der 28-Tages- resp. 90-Tages-Sterblichkeit wurde – wie in der Diskussion ausführlich erläutert – verwendet, da es für die internationale Vergleichbarkeit geeigneter ist als Daten zu Krankenhaus- oder Intensivsterblichkeit. Es ist auch das Standard-Outcome in internationalen Metaanalysen (z. B. Luhr et al. [3]). Umgekehrt sind Studien, die auf international nicht vergleichbaren Outcomes basieren, für einen wie von uns vorgenommenen internationalen Vergleich ungeeignet.

Insbesondere ein Vergleich der Krankenhaussterblichkeit entzieht sich u. E. schon aufgrund der unterschiedlichen Gesundheitssysteme einem internationalen Vergleich; dies zeigt sich auch in den erheblichen Unterschieden bezüglich der Krankenhausverweildauer, die ihrerseits oft ein Auslagern von moribunden Patienten aufgrund von Ressourcenknappheit in „long term facilities“ widerspiegelt.

Somit ist ein Vergleich der Zeit bis zum Versterben (28 Tage) obligat und geeigneter als ein Vergleich in Bezug auf den Ort des Versterbens (Krankenhaus). Nichtsdestotrotz verstehen wir, dass man für die Bewertung innerhalb Deutschlands oder zur Abbildung anderer Ziele auch andere Kennzahlen heranziehen kann. Die der Metaanalyse zugrunde liegenden Forschungsfrage untersuchte die Krankenhausletalität nicht. Daher wird hierüber auch keine Aussage getroffen. Dies stellt aber keine Schwäche unserer Arbeit dar, sondern spiegelt folgerichtig wider, dass nur Antworten auf die gestellten Forschungsfragen gegeben werden – dies war in unserem Fall der Vergleich der 28-Tages-/90-Tages-Letalität über Systemgrenzen und eben nicht ein Vergleich der (hierfür ungeeigneten) Krankenhaussterblichkeit. Unsere Arbeit betrachtet damit etablierte und internationale gebräuchliche Outcomes zur Messung der Sepsissterblichkeit im internationalen Vergleich.

Die Leserbriefe adressieren, dass 4 Quellen (Bloos [4], Bloos [5], Schädler [6], Brunkhorst [7]), in denen nicht zwischen septischem Schock und Sepsis differenziert wird, als septischer Schock ausgewertet werden. Richtig ist, dass diese 4 Studien Patienten mit schwerer Sepsis und septischem Schock untersuchen und der Großteil der Patienten in den Studien an einem septischen Schock erkrankt war (Anteile der Patienten mit septischem Schock: Bloos [4]: 76 %, Bloos [5]: 87 %, Schädler [6]: 94 %, Brunkhorst [7]: 71 %). Die Datenextraktion erfolgte daher in der Form, wie im Protokoll beschrieben. Dass Patientengruppen ausgewertet werden, in denen Teilpopulationen, beispielsweise mit einem anderen Schweregrad, nicht exakt auseinandergehalten werden können, ist unstreitig ein in Metaanalysen etabliertes und weitverbreitetes Vorgehen (beispielsweise [8–13]). Wir stimmen den Leserbriefschreibern aber ausdrücklich zu, dass der Einfluss dieser Unschärfe im Rahmen von Sensitivitätsanalysen untersucht werden sollte. Aus diesem Grund haben wir diese Sensitivitätsanalyse nachgeholt. Die Ergebnisse ändern sich demnach wie folgt: Sensitivitätsanalyse von Studien, die zu 100 % Patienten mit septischem Schock beinhalten: 30-Tages-Mortalität für Deutschland 34,6 % (95 %-KI: 32,90–36,42 %; vgl. mit Hauptanalyse 30,5 % (95 %-KI: 29,30–31,67 %)).

Auch unter dieser Sensitivitätsanalyse zeigt sich jedoch erneut, verglichen mit internationalen Zahlen zur 30-Tages-Mortalität der Metanalysen von Shankar-Hari et al. [14]: 46,5 % (95 %-KI, 42,7–50,3 %) und Vincent et al. [15]: 36,7 % (95 %-KI 32,8–40,8 %), kein Anhaltspunkt, dass die 30-Tages-Sterblichkeit bei septischem Schock im internationalen Vergleich in deutschen Studien erhöht wäre.

Wir danken für den konstruktiven Hinweis, dass bei den Daten von Behnes [16], Bloos [17], Scheer [18] und Simon [19] die Daten für Sepsis nicht separat analysiert wurden. Diese differenzierte Beschreibung des septischen Schocks war in den betreffenden Studien nur in den Supplements enthalten und ergab sich nicht direkt aus den Studien. Es ist daher natürlich wichtig und richtig, diese Daten in unserer Studie zu berücksichtigen, weshalb wir die Metaanalyse wie folgt korrigiert haben: Inkl. der 30-Tages-Sterblichkeiten von 51,0 % (Behnes [16]), 30,7 % (Bloos [17]), 35,0 % (Scheer [18]) und 14,7 % (Simon [19]) ergeben sich folgende Daten: 30-Tages-Sterblichkeit Sepsis in deutschen Studien 26,5 % (95 %-KI 19,86–33,15 %; alt: 22,67 % (95 %-KI: 15,30–30,04 %)) (resp. in oben genannter Sensitivitätsanalyse unter Einschluss der Studien, die auch Patienten mit septischem Schock beinhalten: 26,8 % (95 %-KI: 22,02–31,63 %)).

Wenn man dieses Ergebnis mit der in der Metaanalyse von Stevenson et al. [2] angegebenen 30-Tages-Mortalität von 33,2 % vergleicht, zeigt sich ebenfalls kein Anzeichen für eine erhöhte Sepsisletalität in deutschen Studien. Wir haben daher dazu ein Erratum der Studie veranlasst; die Kernaussage bleibt jedoch unverändert.

Damit möchten wir zum zweiten Themenkomplex, einem Interessenkonflikt, der aus der Vergabe der Analyse an eine Firma (LinkCare Stuttgart) resultiert, Stellung beziehen. Die Affiliationen aller Autoren weisen adäquat die Zugehörigkeit zu akademischen oder kommerziellen Institutionen aus; die Arbeit ist adäquat als Auskopplung der Subgruppe der deutschen Daten aus einer früheren Metaanalyse der Autoren (Bauer [20]), die mit finanzieller Unterstützung von CytoSorbents erstellt wurde, ausgewiesen.

Laut AWMF-Regelwerk Leitlinien sind Interessenkonflikte definiert als „Gegebenheiten, die ein Risiko dafür schaffen, dass professionelles Urteilsvermögen oder Handeln, welches sich auf ein primäres Interesse bezieht, durch ein sekundäres Interesse unangemessen beeinflusst wird“. Hierunter werden berechtigterweise seit geraumer Zeit weit mehr Faktoren als die finanzielle Unterstützung durch eine Firma gesehen. Zu sekundären Interessen zählen daher auch akademische, klinische, persönliche Interessen, deren Ausprägungsgrade und Bedeutungen variieren können. Alle Autoren:innen der Arbeit haben darüber hinaus keinerlei persönliche Interessenkonflikte anzugeben.

Im Gegensatz dazu ist in Bezug auf das zitierte Regelwerk der AWMF zu potenziellen Interessenskonflikten festzustellen, dass die angeblich in Deutschland bestehende Übersterblichkeit septischer Patienten:innen, wie von Reinhart angeführt, zur Forderung nach externen Qualitätssicherungsmaßnahmen herangezogen wird. Begründet wird diese Forderung mit einer, aus Sicht der Autoren, unzureichenden Datenbasis, die ausschließlich auf der Krankenhaussterblichkeit dieser Patienten:innen fußt.

Aus diesem Grund schließen wir uns hier ausdrücklich der Forderung von Gründling an, dass Daten zu diesem Thema auch für die Versorgungssituation in Deutschland und Europa, basierend auf 28-Tage- und 90-Tage-Sterblichkeit, generiert werden sollten.

In Abwesenheit solcher validen Daten sollten Forderungen nach externen Qualitätssicherungsmaßnahmen bei Sepsis, die zu Recht nicht unumstritten sind [21], ausbleiben und bestehende Systeme unter Beachtung der aktuellen Literatur kritisch bewertet werden.
